# Maladaptive evolution or how a beneficial mutation may get lost due to nepotism

**DOI:** 10.1038/s42003-022-03901-z

**Published:** 2022-09-15

**Authors:** Irith Aloni, Amiyaal Ilany

**Affiliations:** Faculty of Life Sciences, Bar Ilan University, Ramat Gan, Israel

**Keywords:** Social evolution, Behavioural ecology

## Abstract

Spotted hyenas are an exception in the animal kingdom not only due to female dominance over males, but also because of the strict female linear hierarchy which determines priority of access to resources and produces considerable female reproductive skew. This special social system raises a question: what would become of a beneficial mutation if it occurred in a low-ranking female? We used several simulation models in order to address this question. Our modeling results indicate that such a social system may inhibit the establishment of a beneficial mutation. However, this negative effect may be counteracted by random choice of mates by females.

## Abstract (web summary)

Evolutionary simulations demonstrate how beneficial mutations may not be preserved in societies characterized by female dominance and social inheritance of rank, such as in spotted hyenas.

## Introduction

Reproductive skew is defined as the uneven distribution of reproductive success among group members of the same sex^1–4^. In species with reproductive skew, variance in reproductive success is a direct consequence of intrasexual competition for breeding and will usually be higher in the sex with the greatest potential rate of reproduction^4,5^. Selection would thus favor traits that improve reproductive success either directly, via intrasexual competition for reproductive opportunities, or indirectly via intrasexual competition for increased social status or access to resources^4,6,7^. Reproductive skew is a common feature among numerous taxa, including a variety of mammalian species^2,3,8^. In the extreme case, only one or two individuals monopolize the entire group reproduction, while others are denied the opportunity to reproduce. Such is the case for honeybees (*Apis mellifera*), Mexican jays (*Aphelocoma ultramarine*), naked mole-rats (*Heterocephalus glaber*), meerkats (*Suricata suricatta*) and dwarf mongooses (*Helogale parvula*) among others^2^.

In mammals, female reproductive output is limited by the number of offspring that can be produced in a single reproductive cycle as well as by the length and energy requirements of gestation and lactation periods. Males, on the other hand, may, hypothetically, sire an almost unlimited number of young, restricted merely by the number of receptive females available. Indeed, in many mammalian species, reproduction is monopolized by one or two males which sire the majority of offspring. Such male reproductive skew exists in species of various mammalian orders including equids, ungulates, rodents, carnivores, and primates^1,3,8–11^.

In social mammals which are organized by a dominance hierarchy, reproductive success is often positively correlated with social rank^1,3,8–14^. In males, social rank is usually determined by physical traits and fighting capabilities^3,9–12,15,16^ (but see Refs. ^17,18^). Yet, reproductive skew is not limited to males. Female reproductive skew is common in many social species, and, as in males, a female’s reproductive success is often positively correlated with its social hierarchical rank^8,9,12,14,19–21^. However, female social rank is often determined by kinship rather than by physical features, particularly in species in which females are philopatric. Young females would thus be usually ranked below their mother^8,11,15,16,19–26^. Moreover, although female reproductive skew may, in some species, be considerable^21,26,27^, unlike males all females usually reproduce (species that employ cooperative breeding such as meerkats (*Suricata suricatta*) or common marmosets (*Callithrix jacchus*)^27^ are an exception to this rule and will not be discussed in this paper).

Nevertheless, when sex roles are reversed, that is, in species in which females are dominant over males, the correlation between reproductive success and female social rank is usually rather weak^25^. This is the case, for instance, in many of the social lemur species^25^. An outstanding exception to this pattern is the spotted hyena (*Crocuta crocuta*), where a highly positive correlation between female social rank and reproductive success has been documented^21,25^. In a long-term study of the spotted hyena in Kenya, Holekamp and Smale^28^ found a five-fold difference in lifetime reproductive success between the alpha female and the lowest ranking one.

Various models have examined the spread of mutations in social species under different conditions including in the case of reproductive skew (e.g., Refs. ^29–32^). McGlothlin and Fisher^33^ used a quantitative genetic model to examine the predicted change in fitness in response to social selection. They showed that strong social selection may lead to maladaptation expressed as a decrease in population mean fitness. However, we were unable to find a model that explores the fate of a beneficial mutation as a function of social status, possibly because social status among males, which are usually the dominant sex as well as the one exhibiting considerable reproductive skew, is determined by physical traits which reflect potential fitness. Under such circumstances, a beneficial mutation which improves the individual’s physical condition would lead to an increased social rank and, thus, would be rather likely to proliferate. But what would become of a beneficial mutation if it appears in a low ranking spotted hyena female, in which social rank is determined merely by nepotism?

In this study we addressed this question using a simulation modeling approach. We created a population dynamic model which imitates the spotted hyenas’ social structure, with a strict female matrilineal nepotistic dominance hierarchy, and immigrant males’ hierarchy underneath that of females^21^. We then induced a beneficial mutation in one of the females and followed its fate along time. Additionally, we followed the fate of two distinct mutations which were applied to one low- and one high-ranking female simultaneously. We demonstrate that a beneficial mutation would be unlikely to become established if occurring in individuals of a low social status.

## Results

### The preliminary *Females only* model

The preliminary model, which included only females, aimed at effectively imitating the spotted hyena social structure and the resulting rank-dependent female reproductive skew. The results of this model, presented in Supplementary Figs. 1–3, indicate a clear reduction in reproductive success with decrease in female social rank. Additionally, in most of the simulations, only one or two of the founding individuals had surviving descendants at the end of the simulation, and those founders usually held an initial rank of three or higher (one being the highest rank). Yet, in a handful of simulations, the initial rank of some founders with surviving descendants was between four and seven. No descendants of any lower-ranking founders ever survived in any of the 200 simulations.

### One mutation models

The one mutation model is an expansion of the preliminary model which consists of five clans (social groups) of males and females. A beneficial mutation was induced in one individual at the beginning of a simulation, and the prevalence of the mutation at the end of the simulation was recorded as well as the social rank of the initial mutant individual. Four different scenarios of male rank and attributes were used. The different scenarios represented a gradually increasing effect of male’s rank and origin on its survival and reproduction probabilities; whereas in Scenario I males lacked any rank and mated randomly, in Scenario IV a male’s rank was determined by both seniority and the male mother’s rank, and male rank in turn affected both death and reproduction probabilities.

**Fig. 1:**
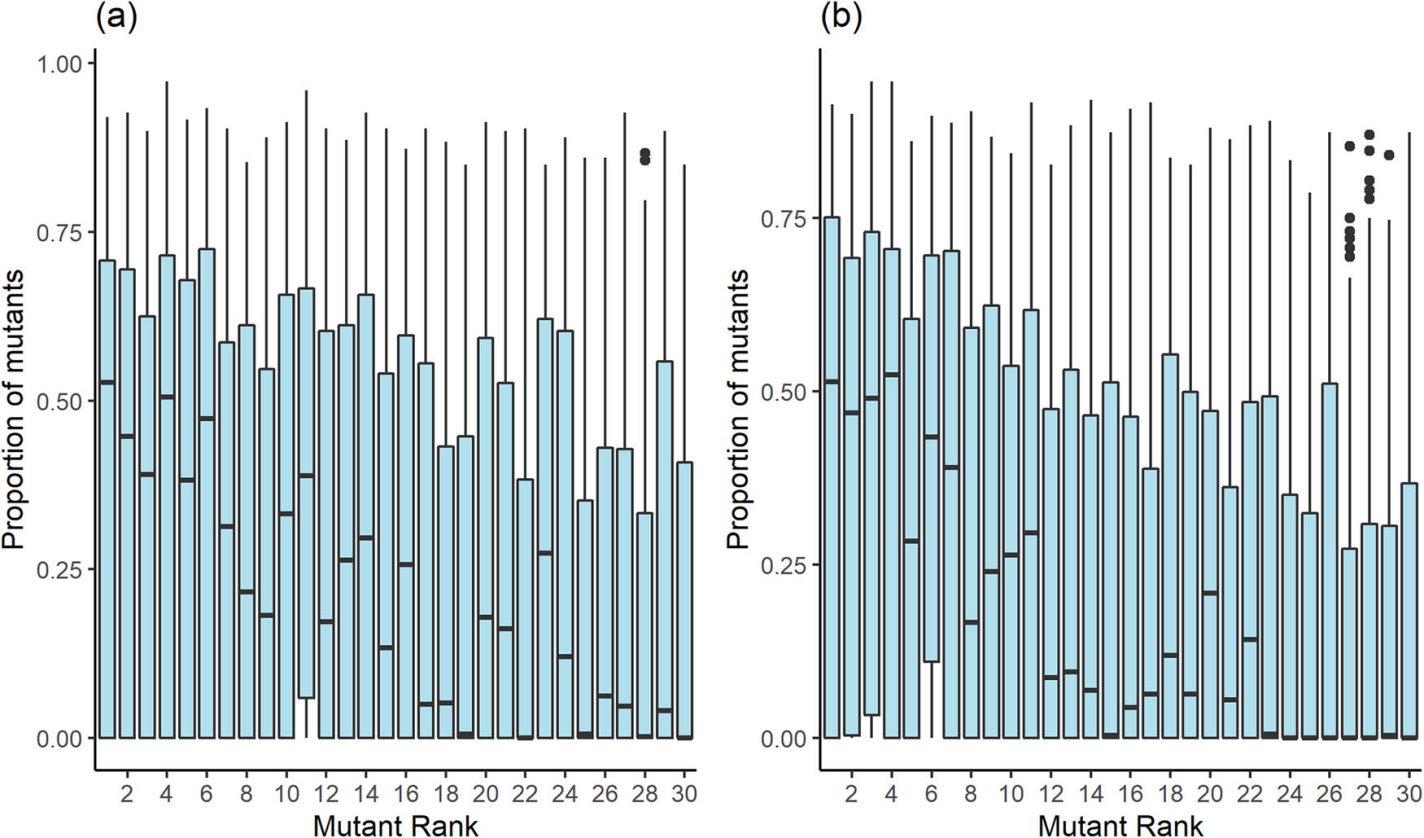
Boxplots of total mutants’ proportion in final population as a function of original mutant female’s rank. **a** Scenario I—no male rank; **b** Scenario II—male’s rank affects death probability. Rank 1 corresponds to highest rank. Population size in all simulations is 300. Each simulation consists of 3000 iterations. 3000 simulations were run for each scenario, 100 simulations for each female’s rank. Boxplots show the interquartile range (box), median (black line), median ± 1.5 IQR (error bars), and outliers (dots).

**Fig. 2:**
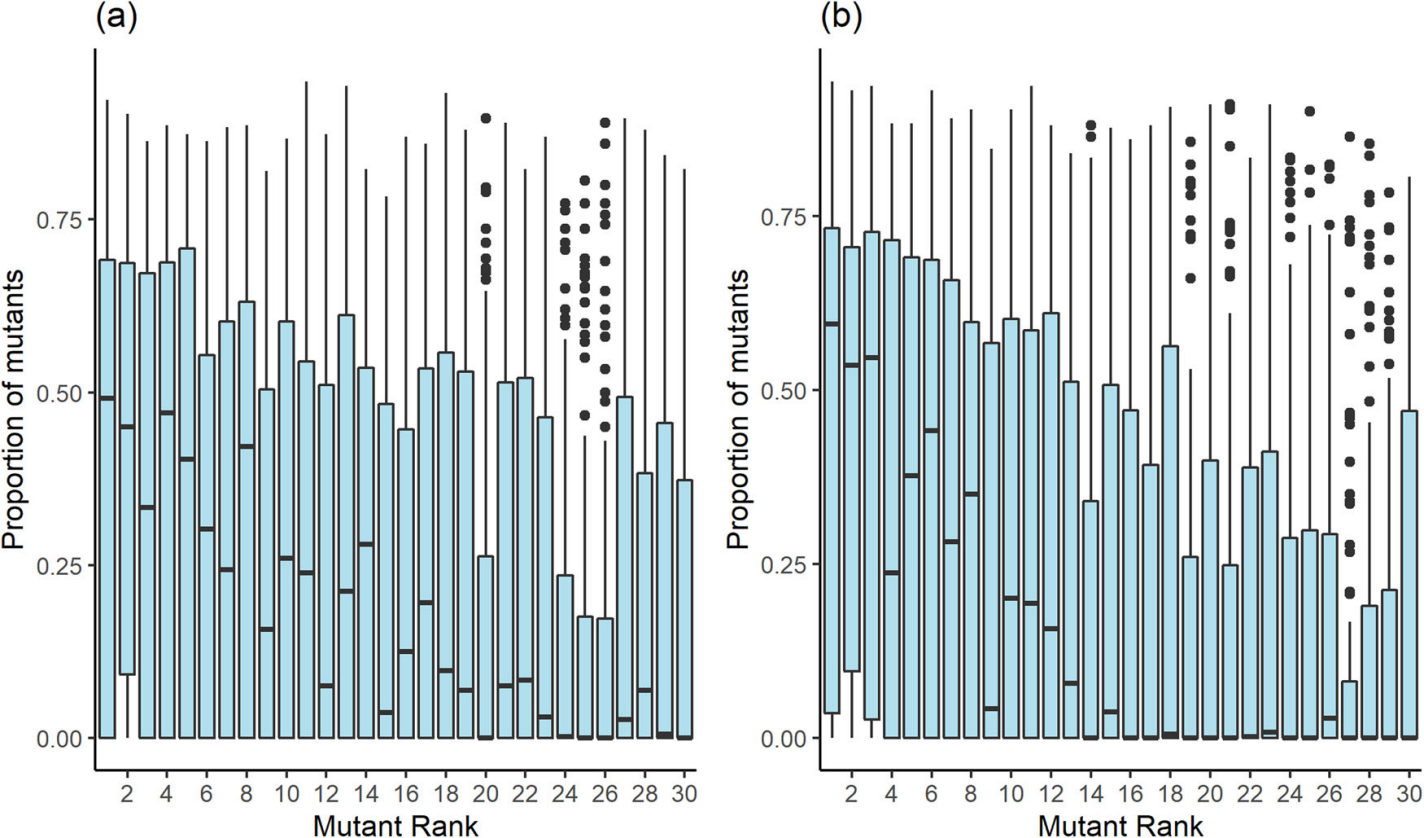
Boxplots of total mutants’ proportion in final population as a function of original mutant female’s rank. **a** Scenario III—male’s rank attained by order of joining clan; **b** Scenario IV—male’s rank depends on mother’s rank. In these two scenarios male’s rank affects both death and reproduction probabilities. Rank 1 corresponds to highest rank. Population size in all simulations is 300. Each simulation consists of 3000 iterations. 3000 simulations were run for each scenario, 100 simulations for each female’s rank. Boxplots show the interquartile range (box), median (black line), median ± 1.5 IQR (error bars), and outliers (dots).

All four models (scenarios) of this stage, simulating the fate of a beneficial mutation along ten generations, resulted in a clear pattern of decrease in mutation establishment rate with decreasing rank of the original mutant female. The median value of mutation establishment rate in all four scenarios was zero for the lowest ranks and above 0.45 for the highest ranks (Figs. 1 and 2, See Supplementary Table 1 for details). However, in the first scenario a considerable mutation establishment is sometimes observed even when originating in a low-ranking female. The median, in this case, is rising quite quickly with increase in rank, getting above 0.1 (i.e., 10% establishment rate) at a rank as low as 24 (Fig. 1a). In Scenario II (Fig. 1b) the establishment rate is essentially a flat zero up to the rank of 22, and except for the ranks of 20 and 18, it remains rather low up to rank 11. In Scenario III (Fig. 2a) the first rank with a median establishment rate > 0.1 is 17, and in Scenario IV (Fig. 2b) the first case of such a median is rank 12.

Similarly, the establishment rate spread as expressed by the interquartile range (*IQR—*the central 50% of distribution presented by the boxes height in the figures) decreases in size and location when moving from high to low ranks, the decline becoming more and more prominent when moving from Scenario I to Scenario IV (Figs. 1 and 2). Interestingly, in Scenario IV, *Q1* (the first quartile) of the three highest ranks is positive, indicating that the mutation in these cases has survived in >75% of the simulations (Fig. 2). Overall, the decrease in mutant frequency with rank of origin gets gradually steeper when moving from Scenario I to Scenario II, to Scenario III, and to Scenario IV.

### Two mutations models

In this stage, two mutations were assigned simultaneously to two different individuals, one of a low rank, the other of a high rank. As before, the fate (prevalence) of each mutation at the end of the simulation, and the rank of the original mutant individual were recorded.

**Fig. 3:**
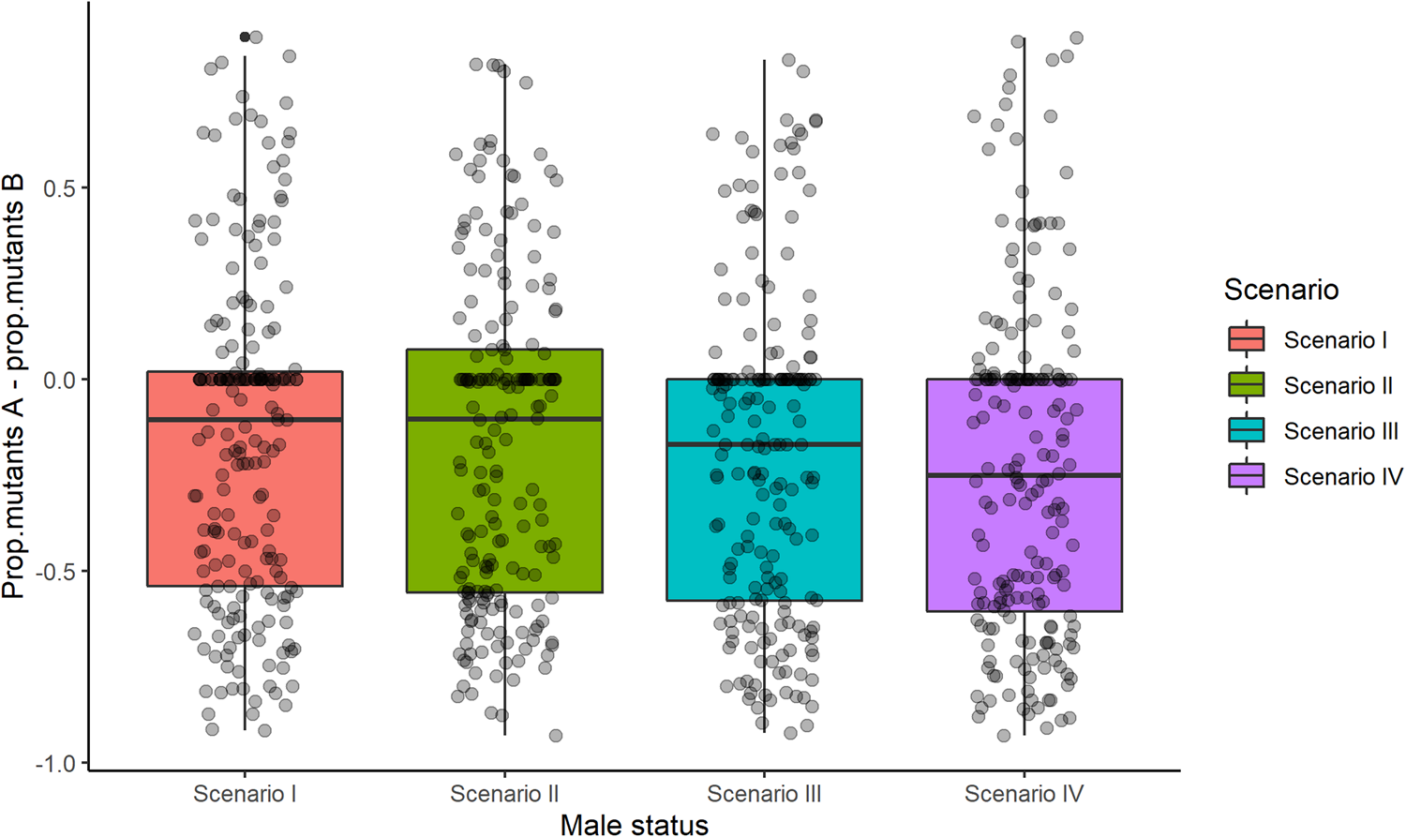
Boxplots of the difference in mutation establishment rate between mutation A (originating in a low rank) and mutation B (originating in a high rank) in the four scenarios of the two mutations model. Population size in all simulations is 300. Each simulation consists of 3000 iterations. 200 simulations were run for each scenario. Boxplots show the interquartile range (box), median (black line), median ± 1.5 IQR (error bars), and outliers (black dots). Gray dots represent actual data points.

Overall, the difference between the low and high rank mutation establishment rates was negative in all four scenarios (Fig. 3), indicating a consistently higher establishment rate of mutation B, originating in a high rank, as compared to mutation A which originated in a low-ranking female. Moreover, the median negative difference increased steadily when moving along the four scenarios, from a value of −0.10 in Scenario I to −0.25 in Scenario IV, indicating an increasing rank effect. Additionally, the box location shifted further down to the negative side, although the upper quartile, *Q3*, did not get below zero due to the relatively large number of cases in which none of the mutations survived.

## Discussion

Our model results indicate that in species with a strict social dominance hierarchy where social rank is determined by nepotism, a beneficial mutation occurring in a low-ranking female is not very likely to get established. This outcome emerged despite the immense advantage of the modeled mutation, which doubled its carrier’s survival probability. Moreover, the reproductive skew in our model (see Supplementary Fig. 1) was less radical than the skew reported for the spotted hyena females^21^, which means that in the model, low-ranking females had a relatively higher reproductive success potential than in reality. In other words, our model may be underestimating the severity of the negative selection a low rank induces.

It is reasonable to assume that a low-ranking mutant female in a female dominant society would produce very few surviving offspring due to her low rank and ensuing lack of access to resources. Thus, this female would have only a slight chance to transmit the mutation to the next generation. If this female does reproduce successfully and produces a female which also inherits the mutation, chances of this daughter to pass on the mutation are also slim, as her rank would be even lower than that of her mother. However, if the young produced is a male and has inherited the mutation, chances of transmitting the mutation may increase depending on the male’s reproduction odds. As demonstrated by the four scenarios, the reduction in mutation establishment with decreasing mutant female’s rank became more and more prominent with increasing restrictions on male reproduction. In all four scenarios, the mutation establishment rate median was zero for the lowest ranking mutants, and in all cases but Scenario I, it was <0.1 for at least the eight lowest ranking mutants (which represent 27% of females; Figs. 1 and 2). Yet, in Scenario I, where all males were equally likely to reproduce and die (except for mutants), the mutation had established rather well in many of the middle ranking mutants, with establishment rate median as high as 0.27 for the low rank of 23 (Fig. 1a). On the other end, in Scenario IV, establishment rate median values of above 0.25 are observed only for ranks of eight or higher, and even values just over 0.1 do not occur before the rank of 12 (Fig. 2b, Supplementary Table 1).

Thus, it follows that in species with female dominance over males and a strict female social hierarchy determined by nepotism, male selection scheme for reproduction may be crucial for the facilitation of a beneficial mutation establishment. Getting back to the spotted hyena, it is the second scenario that best describes this species social structure. In the spotted hyena males are ranked below females, but males’ rank affects only their priority of access to resources (which should affect survival, see^21^), whereas reproduction chances are independent of rank^9,21,26,34^. In fact, some females, particularly young ones, prefer lower-ranking immigrants, presumably to avoid inbreeding^21,25,34^. Since all immigrant males get to be new and low-ranking upon joining the clan, this behavior may be equivalent to giving an equal opportunity to all immigrant males. Indeed, Watts^35^ as well as Höner et al.^26^ found little reproductive skew among immigrant spotted hyena males. This may have two different consequences. On one hand, a male born to a low-ranking female may be less likely to survive due to low access to food in its natal clan and thus a rather poor physical condition upon dispersal, and for the same reason, it may have less chances to be admitted into a new clan^26^. In this case, the mutation would most likely be lost. On the other hand, a mutation may allow a migrating male of a low rank origin to better survive (in our model the mutation multiplied survival probability, a property which may be expressed in various ways, such as, for instance, better hunting capabilities), and thus the mutant male may be more likely to successfully join a new clan (either because its physical condition may permit more persistence upon encountering a new clan, or due to longevity which would facilitate extra search for clans). One way or another, it seems that the female preference for low-ranking males in the spotted hyena, not only serves to prevent inbreeding, as suggested by Höner et al.^34^, but also allows more gene flow and establishment of beneficial mutations, which otherwise would get lost.

Notably, the observed decreasing likelihood of mutation establishment along Scenarios I to III takes place in spite of complete independence between male choice and mutation presence. Male rank in these three scenarios either does not exist (Scenario I) or is defined merely by the order of joining a clan, which is independent of maternal rank or mutation presence. In the two first scenarios, mate choice by females is random. In Scenario III, male rank affects mate choice, but it is still completely independent of mutation presence or maternal rank. The observed diminishing establishment rate of the mutation stems, most likely, from the scarcity of the mutation when originating in a low-ranking female. As long as the mutation is rare, the probability of any single individual carrying it is low, and thus when a few males are more likely to survive and reproduce, the probability that this limited group of males carries the mutation would be further reduced compared to a larger group of potential mates.

The two mutations model further supports our conclusions. The fact that the median difference between the two mutations frequencies was negative in all four scenarios (Fig. 3) undoubtedly implies that a low rank constrains the establishment of a beneficial mutation. Moreover, the entire *IQR*, that is, 75% of the distribution of the difference in mutation establishment rate, rests in the negative region for Scenarios III and IV, and almost so for the first two scenarios. As in the single mutation models, the gradual increase in male reproduction restrictions along the four scenarios is reflected in a shift in location of both the median and the *IQR* of the mutations’ establishment rate difference.

Recent studies have examined the phenomenon of female dominance over males in a variety of species^25,36–39^. Some controversy has arisen regarding the definition of this term. Whereas the traditional definition of dominance examines the results of agonistic interactions and the evoking of submissive behavior^25,36,37,40^, later researchers suggest more subtle definitions such as priority of access to food and seasonal dominance which depends on energetic requirements at various life stages^38–40^. Thus, a variety of species, some of which rarely exhibiting agonistic interactions, or in which such interactions are not always decided one way or another, are included under this subtle definition of female dominance^38–40^. Yet, in most of these species, reproductive skew does not exist or was not reported. One major taxonomic group in which female dominance is the rule is the Lemuridae family, but reproductive skew is very low to nonexistent^25,37,39^. Other species in which some level of female dominance exists include the rock hyrax (*Procavia capensis*), rufous elephant shrew (*Elephantus rufescens*), velvet-furred swamp rat (*Rattus lutreolus*), brush-tailed possum (*Trichosurus vulpecula*), nutria (*Myocastor coypus*), Peruvian squirrel monkeys (*Saimiri boliviensis peruviensis*), Columbian ground squirrels (*Spermophilus columbianus*), snowshoe hares (*Lepus americanus*), Dall’s sheep (*Ovis dalli*), Angolan talapoin (*Cercopithecus talapoin*), rhesus macaque (*Macaca mulata*), tufted capuchin (*cebus apella*), bonobos (*Pan paniscus*), Garnett’s greater bushbabies (*Otolemur garnettii*), golden and Chinese hamsters (*Mesocricetus, Cricetulus spp*.), Maxwell’s duikers (*Cephalophus maxwelli*), otters (*Amblonyx cinerea, Lutrogale perspicilla*), and beavers (*Castor canadensis*)^25,36,38,39,41^. Although female dominance hierarchy exists in a few of these species (e.g., Peruvian squirrel monkey^41^, ring-tailed lemur (*Lemur catta*)^39,42^, Verreaux’s sifaka (*Propithecus verreauxi*))^13,25^, we did not find any studies indicating female reproductive skew in any of them. Holekamp and Engh^25^, who reviewed the more classical female dominant species, also reported no evidence for female reproductive skew.

This seemingly lack of female reproductive skew among most female dominant species is quite surprising in light of the rather common correlation between social rank and female reproductive success in male dominant species. To mention a few, considerable female reproductive skew is found in baboons (*Papio spp*), macaques (*macaca spp*.), feral horses (*Equus caballus*) and plains zebras (*Equus burchelli*)^8,15,19^.

Holekamp and Smale^28^ state that “reproductive skew among female spotted hyenas appears to be greater than that documented among females of male-dominated species characterized by plural breeding”. They suggest that the key determinant of reproductive success among females in this species is rank-related priority of access to food resources. This high priority is reinforced by female dominance over males and is particularly important as this species resides in an environment in which prey availability is seasonal and scarce at times^21^. Our study suggests that this extreme difference in reproductive success, which, unlike in male-dominated species, is determined by nepotism rather than by physical characters, may induce a handicap on the entire population preventing the establishment of beneficial mutations. This may also hinder adaptation to a changing environment. However, our study results indicate that male equal access to females may, at least partially, counter the inhibition effect on a beneficial mutation establishment. More research is necessary in order to investigate female reproductive skew in species with a social structure similar to that of the spotted hyena, which is characterized by female dominance over males, plural breeding, and a strict female linear social hierarchy determined by nepotism.

One intriguing possibility for testing this model’s validity would be an empirical study, provided that the value of some adaptive trait can be measured. In the case of the spotted hyena such a trait may refer to hunting success or physical capabilities. It is well established that adult female spotted hyenas are larger and more aggressive than adult males^21^, but little attention has been allocated to the study of individual physical differences among females of different ranks. Smith et al.^43^ studied within clan aggression in the context of the fission-fusion behavior characterizing the spotted hyena clans. Their results indicate more frequent aggression and resulting fissions occurring during times of food shortage. Rank was found to be the major correlate of an aggressive incident result. If it is possible to identify low-ranking females with some beneficial trait (independent of rank), it would be interesting to follow such females’ inclusive reproductive success along time, and even more so, the reproductive success of their sons.

Another possible path around the conflict this model suggests would be through the selection of male admission into new clans. Male admission into clans is often constrained by severe aggression of resident immigrant males which may prevent or delay male admission^21,26^. Such behavior may in fact promote mutant male chances, at least in the case of a mutation that improves physical capabilities.

One last, though not very likely possible detour around this difficulty is the occurrence of dominance rank exchanges. Such rank improvements are not very common among female dominated societies, except for in the case of aging females who may clear the way for their daughters^44^. However, Straus and Holekamp^44^ found that individuals who repeatedly form coalitions with their top allies are likely to improve their position, and, according to Strauss and Holekamp^44^, “facilitate revolutionary social change”. It should be kept in mind that not only are such incidents rather rare, but they are unlikely to turn a very low-ranking female into a high-ranking one, especially not when group size is large.

More empirical and theoretical research should shed more light on this intriguing question of possible maladaptive evolution. Our model, in line with a few other models such as that of Holman^31^, suggests that evolution may not always lead to the best solution. As in every process, a local optimum may get evolution trapped and prevent further advance to better optima.

## Methods

The social structure of the spotted hyenas served as a framework for our model. The basic social unit of the spotted hyena, called a clan, consists of several matrilines of natal females and their offspring, and some immigrant males. Clan members are organized in a strict linear hierarchical structure which, for natal individuals, is determined by kinship, where juveniles acquire a rank immediately below their mothers^9,21,25,28^. Immigrant males get their rank by order of joining the clan and all females are dominant to all immigrant males. Dominance rank determines priority of access to resources, particularly food^9,21,25,26,28,35^. Natal males usually disperse upon sexual maturation^21^. Mate choice is employed by females, which are physically larger than males, and is not affected by rank of either male or female^9,21,25,26,28,35^. Rank-dependent priority of access to food shapes female’s physiological state which in turn affects rate of reproduction and longevity. Additionally, due to food access priority, growth rate of cubs of high-ranking females would be accelerated compared to low-ranking ones^21,25,26^. These rank effects are probably the cause of the large reproductive skew observed among spotted hyena females^21,25,26,45^.

Modeling consisted of three stages: The first stage was a preliminary one aimed at creating a model that effectively imitates the spotted hyena social structure and the resulting reproductive skew. For this purpose, we modeled one clan female-only dynamics, as the presence of adult males is irrelevant to female social hierarchy structure and reproductive skew^9,21,25,28,35^.

After reaching a model that successfully imitated female reproductive skew, we expanded the model to include a population of five clans and incorporated males. We then added a beneficial mutation to one of the females and followed the mutation fate along time. In the third stage, we used the later model and assigned two independent beneficial mutations on two different genes. One mutation was assigned to a low-ranking female, the other to a high-ranking female. Both mutations had the same effect. The fate of these two simultaneous mutations was followed over time.

### The preliminary model

Our model is an agent-based model^46^. The initial *Females only* model consists of 50 individual females arranged in a linear social hierarchy. On each iteration, one individual dies and one gives birth to a single newborn. The individual probabilities of death and birth are functions of social rank, with increasing probability of death and decreasing probability of reproduction with decreasing social rank (see Supplementary Note 1 for more details). A newborn receives a social rank right below its mother. We ran the model for 500 iterations representing ten generations and repeated the procedure in 200 simulations. Reproductive success of the final generation females as a function of their initial social rank (rank upon birth), and the ranks of founding females’ matrilines surviving to the end of a simulation were recorded.

### One mutation models

In the second stage the preliminary model was expanded to include 5 clans, each of them consisting of an initial population of 30 females and 30 mature unrelated males (immigrants), males being ranked under all females in a linear hierarchy. On each iteration, a clan was selected randomly. In the selected clan, one individual died according to a rank-dependent probability function, and a female was selected for reproduction according to a rank-dependent reproduction probability. Additionally, a male was selected for reproduction out of the clan’s males (more details below). The newborn was randomly sexed. If a female, it joined its natal clan and was ranked below its mother. If a male, it was moved randomly to one of the four other clans and was ranked at the bottom of hierarchy in that clan.

At the beginning of the simulation, all individuals were assigned a gene with two identical neutral alleles, except for one female which was assigned one neutral allele and one mutant allele. The mutant female was selected for reproduction in the very first iteration. We applied the mutation to different ranks on different simulations. The mutation doubled survival probability and was a dominant one (i.e., a heterozygous individual was affected by the mutation as much as a homozygous individual). A newborn inherited one random allele of the gene from each parent.

Each simulation was run for 3000 iterations, imitating ten generations (the overall population size was 300; 5 clans × 60 individuals). The mutation was assigned to each female’s rank in 100 simulations, summing up to a total of 3000 simulations (30 female initial ranks × 100) which were run for each of four male selection scenarios (see below). At the end of each simulation, we counted the total number of individuals carrying the mutation. This value divided by the total population size was defined as the mutation establishment rate.

The death probability of individual *i* was weighted by the following formula:

1 $${{Die}}_{i}={N}_{{clan}}+{{Rank}}_{i}$$

where: *Rank*_*i*_∈(1…*N*_*clan*_), 1 being the highest rank

*N*_*clan*_ = number of individuals in clan

For a mutant individual, the weight for the death probability was reduced to:

2 $${{Die}}_{i}=0.5({N}_{{clan}}+{{Rank}}_{i})$$

Thus, *p(D*_*i*_*)*, the death probability of individual *i*, was calculated using the following formula:

3 $${p}(D_{i})=\frac{{{Die}}_{i}}{\mathop{\sum }\limits_{j=1}^{{N}_{{clan}}}{{Die}}_{j}}$$

See Supplementary Fig. 4 for an illustration of the mutation effect on death probability.

The reproduction probability weight of females was:

4 $${{Rep}}_{i}={N}_{{clan}}-{{Rank}}_{i}$$

Thus, *p(Bf*_*i*_*)*, the reproduction probability of female i, was calculated as follows:

5 $${p}({Bf}_{i})=\frac{{{Rep}}_{i}}{\mathop{\sum }\limits_{j=1}^{{N}_{{females}}}{{Rep}}_{j}}$$

where *N*_*females*_ is the total number of females in the clan.

Four scenarios of male selection for reproduction were analyzed.

**Scenario I**. No male rank. A male was selected randomly for reproduction, independent of rank. Additionally, death probability was identical for all individuals but the mutants, whose death probability was halved compared to non-mutants. In this scenario, rank did not affect death probability in either males or females.

**Scenario II**. Random male choice for reproduction. Death probability was dependent on rank for all clan members.

**Scenario III**. Choice of male depends on male’s rank. The probability for male reproduction was weighted by:

6 $${{Rep}}_{i}={1.5N}_{{clan}}-{{Rank}}_{i}$$

Thus, *p(Bm*_*i*_*)*, the reproduction probability of male i, was calculated as follows:

7 $${p}({Bm}_{i})=\frac{{{Rep}}_{i}}{\mathop{\sum }\limits_{j=1}^{{N}_{{males}}}{{Rep}}_{j}}$$

where *N*_*males*_ is the total number of males in the clan.

**Scenario IV**. Choice of male depends on male’s rank, and male’s rank is affected by maternal rank. In this case, a male joining a new clan was ranked below the male whose rank among the local males was identical to the newcomer mother’s rank. If no such rank existed (i.e., mother’s rank was lower than total number of males), the new male was ranked at the bottom of hierarchy. The weight for male reproduction probability was identical to that of Scenario III.

The motivation for this last scenario is twofold. First, immigration of spotted hyena into a new clan is not automatic. Male physical state upon emigration, which is considerably affected by its mother’s rank, would affect its probability to survive and successfully join a new clan. Second, this scenario serves to model species in which male rank is determined by means other than order of arrival.

### Two mutations models

In the third stage of our analysis, we ran the above model with two simultaneous mutations which were assigned on two separate genes, one mutation to each of two different individuals. The first mutation, entitled Mutation A, was assigned to one allele of Gene 1 of a low-ranking female. The second mutation, entitled Mutation B, was assigned to one allele of Gene 2 of a high-ranking female. Both mutations had the same effect, halving the weight of the death probability. The reason behind the use of two separate genes was the prevention of possible interaction resulting from direct competition between the two mutations. That is, if both mutations would have been applied to a single gene, an individual heterozygotic to the mutations (i.e., carrying two mutant alleles originating from different sources) could have transmitted only one of these alleles at a time, eliminating the other allele, whereas if the mutations reside on separate genes, both mutations could be inherited independently.

As before, each gene consisted of two alleles, all neutral at the simulation starting point. In the first iteration, Mutation A was assigned randomly to one of the lowest-ranking females of ranks 24–30. On the second iteration of each simulation, Mutation B was assigned randomly to one of the highest-ranking females of ranks 1–6. The effect of both mutations was identical to the effect of the mutation in stage II, doubling the probability of survival. As before, mutations were dominant, and their effect was not intensified if more than one mutant allele was present. Additionally, the presence of mutations on both genes did not change the effect.

Each model was run for 3000 iterations, and each of the four male selection scenarios was run for 200 simulations. The total number of individuals carrying each of the mutations by the end of the simulation was recorded. The final measure used was the difference in establishment rate between Mutation A (originating in a low rank) and Mutation B (originating in a high rank).

### Reporting summary

Further information on research design is available in the Nature Research Reporting Summary linked to this article.

## Supplementary information

Supplementary Information

Description of Additional Supplementary Files

Supplementary Data 1

Reporting Summary

## Data Availability

Simulation results underlying main figures are presented in Supplementary Data 1.

## Code availability

All modeling was done using R software (Version 4.0.4)^47^. The R code used for this work can be found at: 10.5281/zenodo.6906922.
