# Supplementary material for: Maladaptive evolution or how a beneficial mutation may get lost due to nepotism
Source: Commun Biol. 2022 Sep 15;5:965. doi: 10.1038/s42003-022-03901-z (PMC9477802; doi:10.1038/s42003-022-03901-z)
Supplement: Supplementary file 1 — Supplementary Information [file 42003_2022_3901_MOESM1_ESM.pdf]

**Supplementary Note 1**

**Preliminary Model: Female only model**

50 females ranked in a strict linear hierarchy.

*Rank 1* is the highest rank.

Weight for reproduction probability: $Rep_i = 1.5N - Rank_i$

Weight for death probability: $Die_i = N + Rank_i$

where: $N$= population size; $Rank_i$= rank of individual $i$

200 simulations

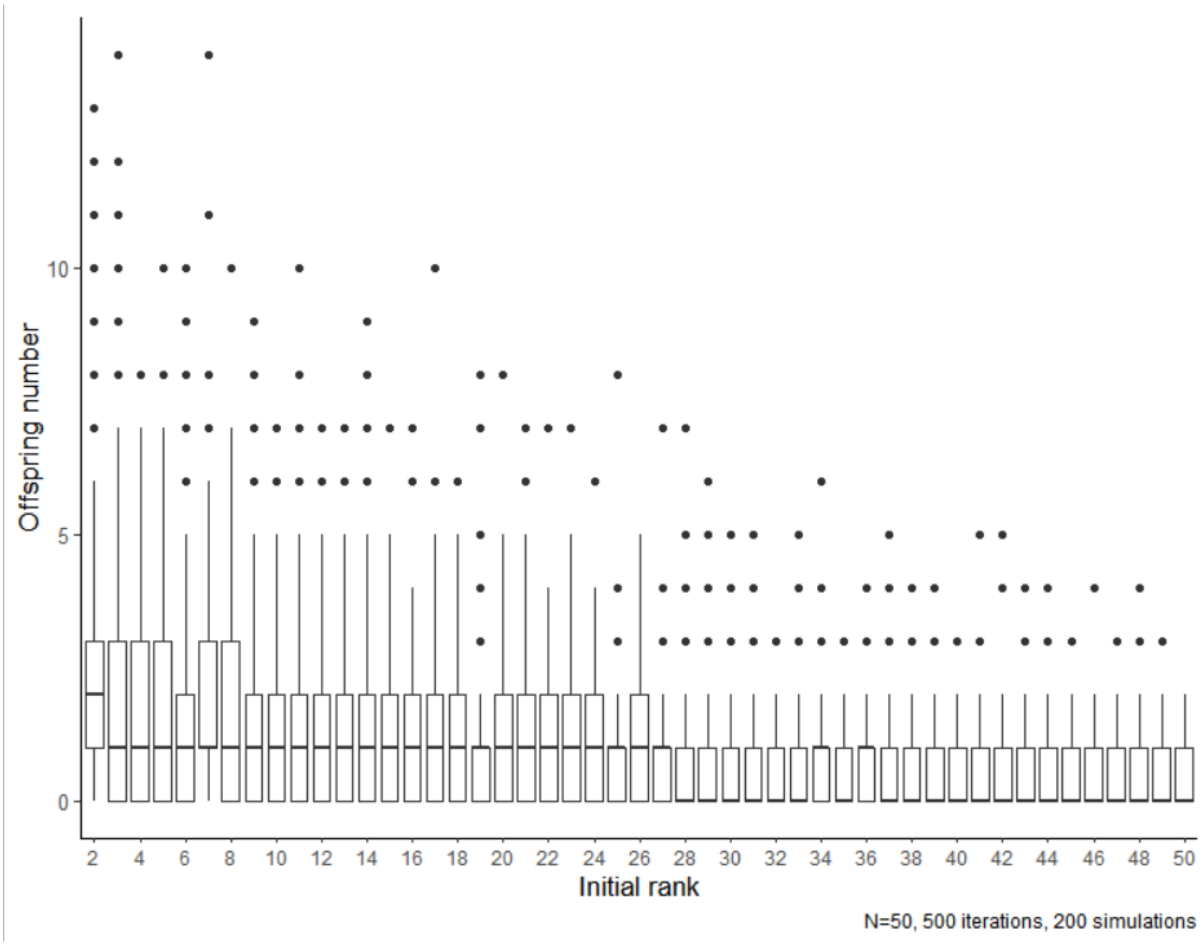


**Supplementary Figure 1.** Preliminary model: boxplots of individuals' offspring number by their initial rank.
Only the 40 oldest individuals (out of 50) at the end of a simulation were used for this graph, as younger individuals may not have lived long enough to reproduce.
Boxplots show the interquartile range (box), median (black line), median ±1.5 IQR (error bars), and outliers (dots).

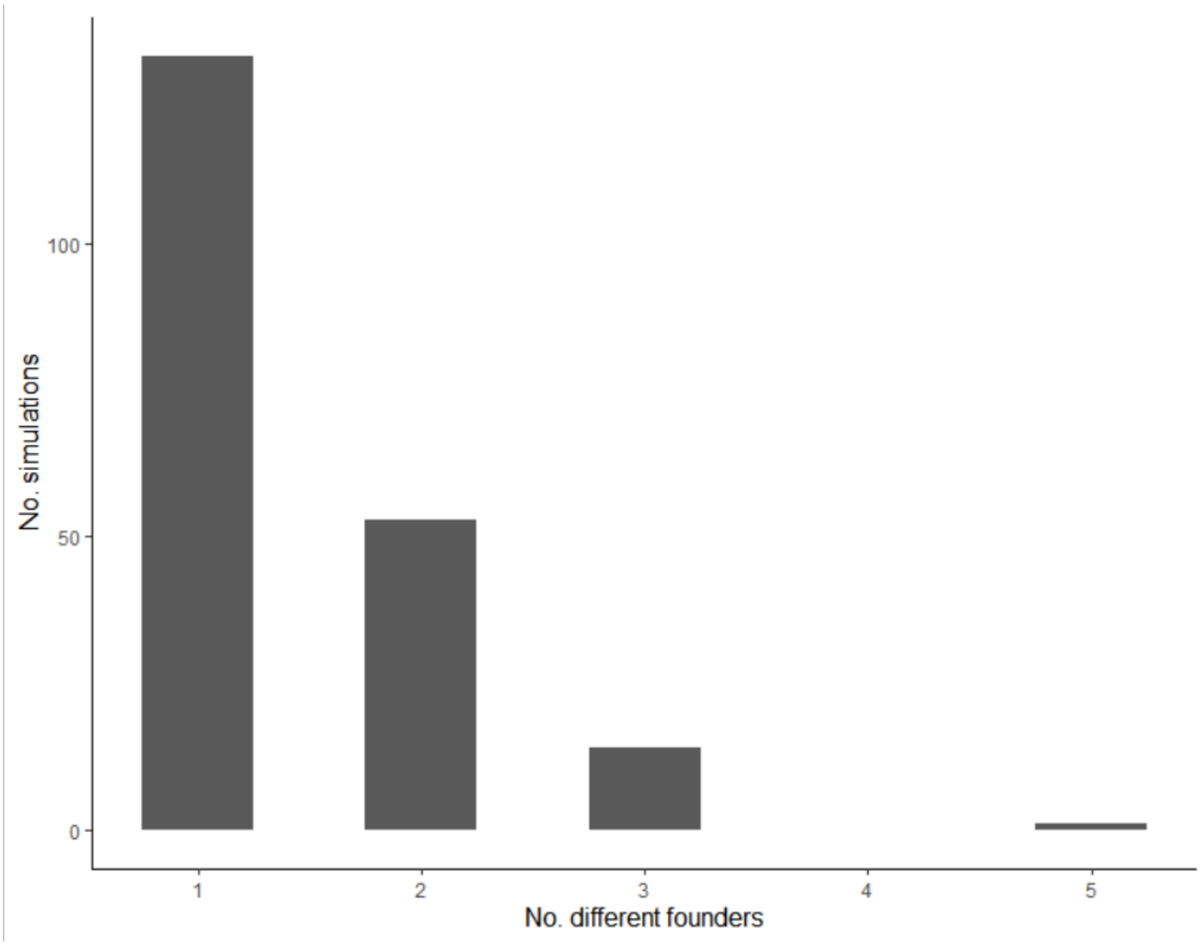


**Supplementary Figure 2.** Preliminary model: bar plot of the number of founder female matrilines surviving at the end of simulations. The height of each bar represents the number of simulations in which this number of different founder matrilines survived.

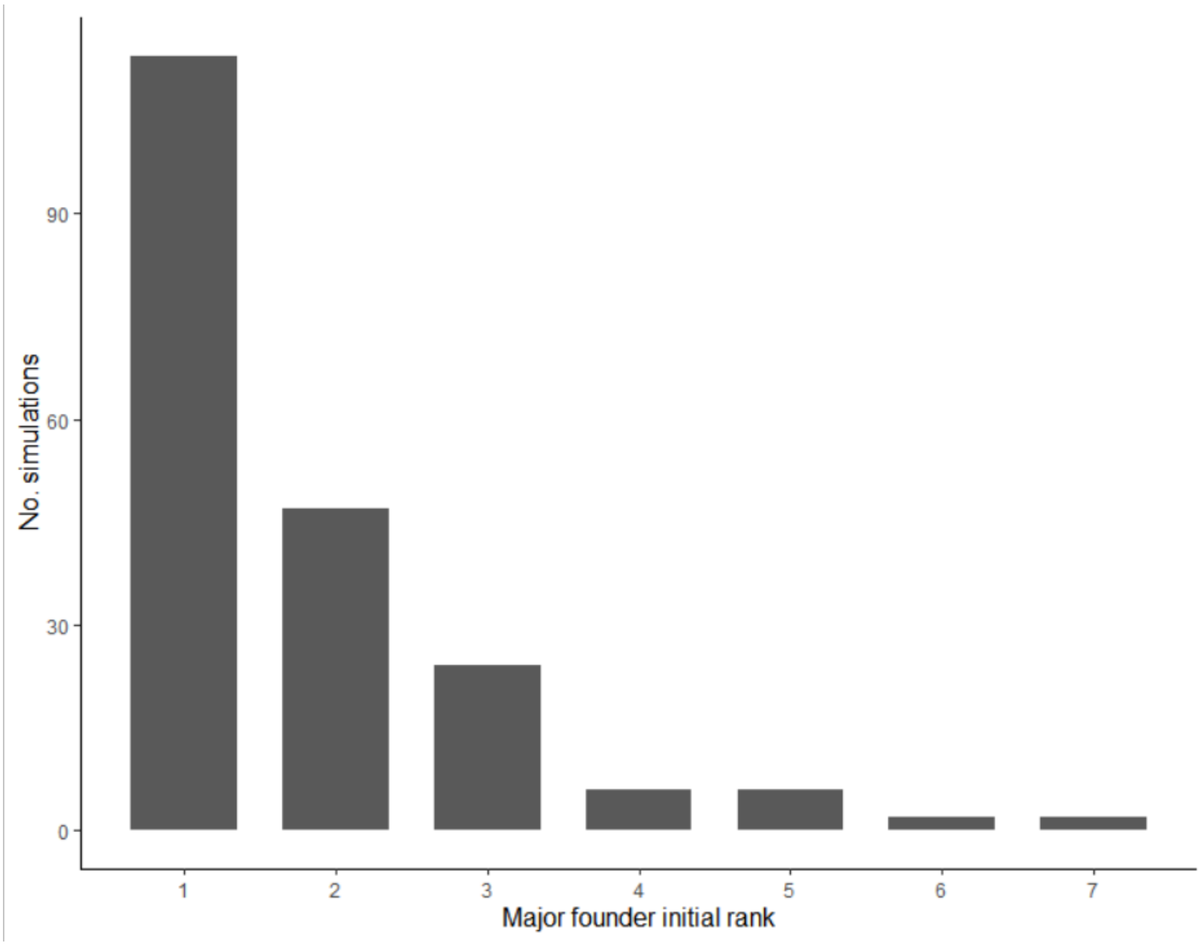


**Supplementary Figure 3.** Preliminary model: bar plot of the initial rank of the major matriline founder in the different simulations.

**Supplementary Table 1.** One mutation model: medians and quartiles of one mutation establishment rate by founder mutant rank for the four scenarios. 100 simulations were run for each founder mutant rank within each scenario

| Mutant rank | Sceanrio I | | | Scenario II | | | Scenario III | | | Scenario IV | | |
|---|---|---|---|---|---|---|---|---|---|---|---|---|
| | Median | Q1 | Q3 | Median | Q1 | Q3 | Median | Q1 | Q3 | Median | Q1 | Q3 |
| 1 | 0.527 | 0 | 0.708 | 0.513 | 0 | 0.751 | 0.492 | 0 | 0.692 | 0.595 | 0.035 | 0.733 |
| 2 | 0.447 | 0 | 0.694 | 0.468 | 0.003 | 0.692 | 0.450 | 0.092 | 0.687 | 0.535 | 0.096 | 0.705 |
| 3 | 0.390 | 0 | 0.625 | 0.490 | 0.033 | 0.729 | 0.333 | 0 | 0.673 | 0.547 | 0.026 | 0.727 |
| 4 | 0.505 | 0 | 0.715 | 0.523 | 0 | 0.705 | 0.470 | 0 | 0.688 | 0.237 | 0 | 0.715 |
| 5 | 0.382 | 0 | 0.678 | 0.283 | 0 | 0.604 | 0.403 | 0 | 0.708 | 0.377 | 0 | 0.690 |
| 6 | 0.473 | 0 | 0.725 | 0.433 | 0.110 | 0.696 | 0.302 | 0 | 0.554 | 0.442 | 0 | 0.687 |
| 7 | 0.313 | 0 | 0.587 | 0.390 | 0 | 0.702 | 0.243 | 0 | 0.603 | 0.282 | 0 | 0.658 |
| 8 | 0.217 | 0 | 0.612 | 0.167 | 0 | 0.591 | 0.422 | 0 | 0.631 | 0.350 | 0 | 0.598 |
| 9 | 0.182 | 0 | 0.547 | 0.240 | 0 | 0.623 | 0.157 | 0 | 0.504 | 0.042 | 0 | 0.568 |
| 10 | 0.332 | 0 | 0.657 | 0.263 | 0 | 0.537 | 0.260 | 0 | 0.603 | 0.200 | 0 | 0.602 |
| 11 | 0.388 | 0.059 | 0.666 | 0.295 | 0 | 0.617 | 0.238 | 0 | 0.544 | 0.193 | 0 | 0.586 |
| 12 | 0.172 | 0 | 0.603 | 0.087 | 0 | 0.474 | 0.075 | 0 | 0.511 | 0.157 | 0 | 0.610 |
| 13 | 0.263 | 0 | 0.612 | 0.095 | 0 | 0.531 | 0.212 | 0 | 0.612 | 0.078 | 0 | 0.512 |
| 14 | 0.297 | 0 | 0.657 | 0.068 | 0 | 0.465 | 0.280 | 0 | 0.536 | 0 | 0 | 0.340 |
| 15 | 0.133 | 0 | 0.540 | 0.003 | 0 | 0.513 | 0.037 | 0 | 0.483 | 0.037 | 0 | 0.507 |
| 16 | 0.257 | 0 | 0.597 | 0.043 | 0 | 0.463 | 0.125 | 0 | 0.446 | 0 | 0 | 0.471 |
| 17 | 0.050 | 0 | 0.555 | 0.063 | 0 | 0.388 | 0.195 | 0 | 0.534 | 0 | 0 | 0.392 |
| 18 | 0.052 | 0 | 0.432 | 0.118 | 0 | 0.553 | 0.097 | 0 | 0.558 | 0.005 | 0 | 0.563 |
| 19 | 0.005 | 0 | 0.447 | 0.063 | 0 | 0.499 | 0.068 | 0 | 0.530 | 0 | 0 | 0.260 |
| 20 | 0.178 | 0 | 0.593 | 0.208 | 0 | 0.472 | 0 | 0 | 0.263 | 0 | 0 | 0.398 |
| 21 | 0.162 | 0 | 0.526 | 0.055 | 0 | 0.362 | 0.075 | 0 | 0.514 | 0 | 0 | 0.248 |
| 22 | 0 | 0 | 0.383 | 0.142 | 0 | 0.484 | 0.083 | 0 | 0.521 | 0.002 | 0 | 0.388 |
| 23 | 0.273 | 0 | 0.621 | 0.005 | 0 | 0.493 | 0.030 | 0 | 0.464 | 0.008 | 0 | 0.411 |
| 24 | 0.120 | 0 | 0.603 | 0 | 0 | 0.350 | 0.002 | 0 | 0.235 | 0 | 0 | 0.287 |
| 25 | 0.005 | 0 | 0.352 | 0 | 0 | 0.324 | 0 | 0 | 0.175 | 0 | 0 | 0.298 |
| 26 | 0.062 | 0 | 0.430 | 0 | 0 | 0.511 | 0 | 0 | 0.173 | 0.028 | 0 | 0.293 |
| 27 | 0.047 | 0 | 0.428 | 0 | 0 | 0.273 | 0.027 | 0 | 0.493 | 0 | 0 | 0.081 |
| 28 | 0.002 | 0 | 0.333 | 0 | 0 | 0.308 | 0.068 | 0 | 0.383 | 0 | 0 | 0.189 |
| 29 | 0.040 | 0 | 0.558 | 0.003 | 0 | 0.306 | 0.005 | 0 | 0.455 | 0 | 0 | 0.213 |
| 30 | 0 | 0 | 0.408 | 0 | 0 | 0.367 | 0 | 0 | 0.373 | 0 | 0 | 0.469 |

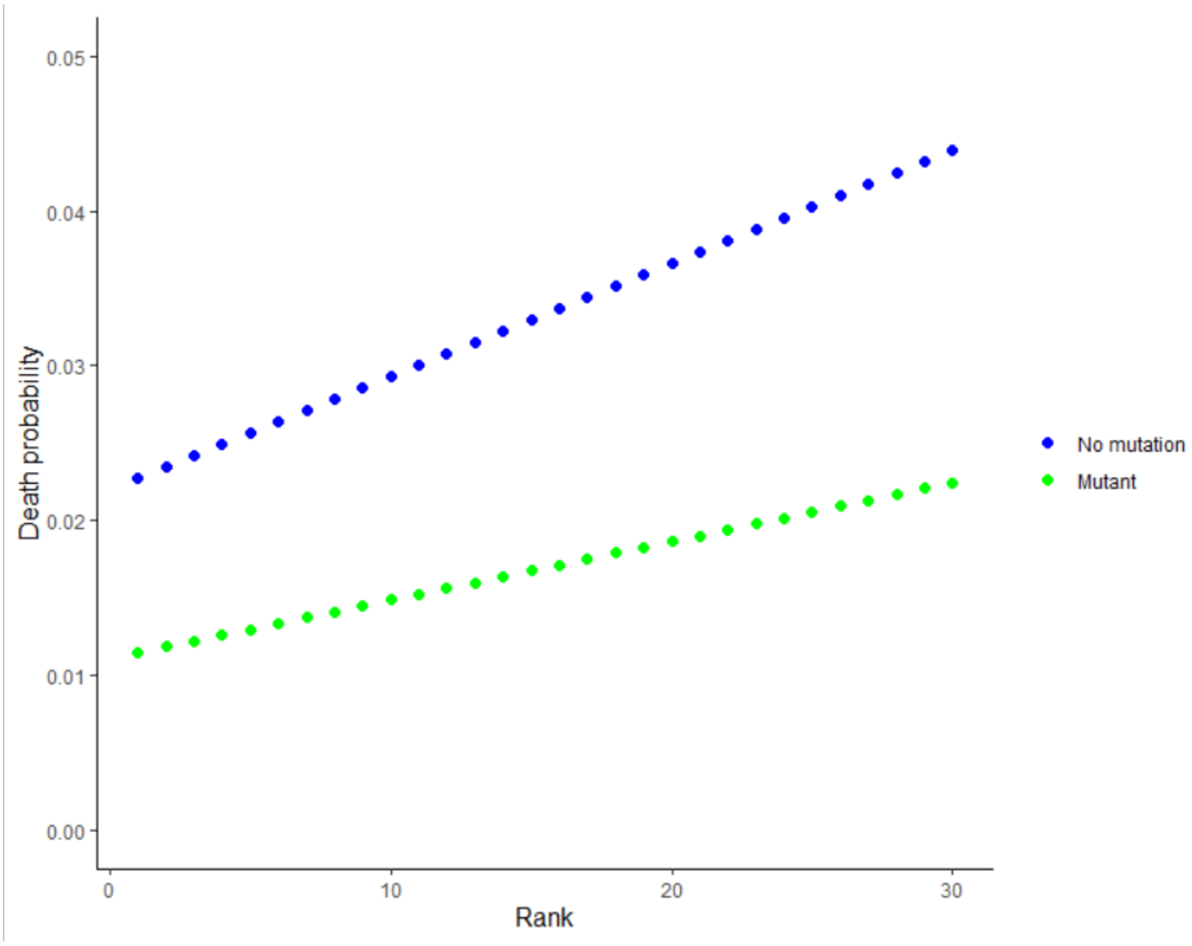


**Supplementary Figure 4.** An example of the effect of a mutation on death probability in a clan of size 30 with a strict dominance hierarchy. The blue dots represent individual death probability as a function of social rank in the case of complete lack of mutations. The green dots represent a mutant individual death probability if it was the only individual to carry the mutation. Notice that the exact values may change if more than one mutant is present.
