# Supplementary material for: Maladaptive evolution or how a beneficial mutation may get lost due to nepotism
Source: Commun Biol. 2022 Sep 15;5:965. doi: 10.1038/s42003-022-03901-z (PMC9477802; doi:10.1038/s42003-022-03901-z)
Supplement: Supplementary file 2 — Description of Additional Supplementary Files [file 42003_2022_3901_MOESM2_ESM.pdf]

# Description of Additional Supplementary Files

**File name:** Supplementary Data 1
**Description:** Simulation results underlying main figures.
